# Autochthonous Peruvian Natural Plants as Potential SARS-CoV-2 M^pro^ Main Protease Inhibitors

**DOI:** 10.3390/ph16040585

**Published:** 2023-04-13

**Authors:** Maria Nuria Peralta-Moreno, Vanessa Anton-Muñoz, David Ortega-Alarcon, Ana Jimenez-Alesanco, Sonia Vega, Olga Abian, Adrian Velazquez-Campoy, Timothy M. Thomson, José Manuel Granadino-Roldán, Claudia Machicado, Jaime Rubio-Martinez

**Affiliations:** 1 Department of Materials Science and Physical Chemistry, University of Barcelona, and the Institut de Recerca en Quimica Teorica i Computacional (IQTCUB), 08028 Barcelona, Spain; 2 Facultad de Farmacia y Bioquímica, Universidad Nacional Mayor de San Marcos, Jr. Puno 1002, Lima 15001, Peru; 3 Institute of Biocomputation and Physics of Complex Systems (BIFI), Joint Unit GBsC-CSIC-BIFI, Universidad de Zaragoza, 50018 Zaragoza, Spain; 4 Departamento de Bioquímica y Biología Molecular y Celular, Universidad de Zaragoza, 50009 Zaragoza, Spain; 5 Instituto de Investigación Sanitaria de Aragón (IIS Aragon), 50009 Zaragoza, Spain; 6 Centro de Investigación Biomédica en Red en el Área Temática de Enfermedades Hepáticas Digestivas (CIBERehd), 28029 Madrid, Spain; 7 Institute of Molecular Biology of Barcelona (IBMB-CSIC), 08028 Barcelona, Spain; 8 Laboratorio de Investigación Traslacional y Biología Computacional, Facultad de Ciencias y Filosofía-LID, Universidad Peruana Cayetano Heredia, Av. Honorio Delgado 430, Lima 15102, Peru; 9 Departamento de Química Física y Analítica, Facultad de Ciencias Experimentales, Universidad de Jaén, Campus “Las Lagunillas” s/n, 23071 Jaén, Spain

**Keywords:** SARS-CoV-2 main protease, Peruvian natural plants, docking, molecular dynamics, MM-PB/GBSA approach, drug design, allosteric inhibition, Hyperoside

## Abstract

Over 750 million cases of COVID-19, caused by the Severe Acute Respiratory Syndrome Coronavirus 2 (SARS-CoV-2), have been reported since the onset of the global outbreak. The need for effective treatments has spurred intensive research for therapeutic agents based on pharmaceutical repositioning or natural products. In light of prior studies asserting the bioactivity of natural compounds of the autochthonous Peruvian flora, the present study focuses on the identification SARS-CoV-2 M^pro^ main protease dimer inhibitors. To this end, a target-based virtual screening was performed over a representative set of Peruvian flora-derived natural compounds. The best poses obtained from the ensemble molecular docking process were selected. These structures were subjected to extensive molecular dynamics steps for the computation of binding free energies along the trajectory and evaluation of the stability of the complexes. The compounds exhibiting the best free energy behaviors were selected for in vitro testing, confirming the inhibitory activity of Hyperoside against M^pro^, with a *K_i_* value lower than 20 µM, presumably through allosteric modulation.

## 1. Introduction

COVID-19, a highly infectious disease caused by Severe Acute Respiratory Syndrome Coronavirus 2 (SARS-CoV-2), first identified in 2019 in Wuhan (China) [1], has demonstrably infected more than 750 million people since the onset of the global pandemic situation declared by the World Health Organization on March 2020 [2,3]. Enormous prevention efforts, including social distancing, regular use of masks and hand washing, have not prevented the spread of the virus [4]. Motivated by the rapid increase in cases and severe illness, much effort has been placed on the discovery of antivirals, by resorting to natural compounds or through drug repositioning strategies [5,6,7,8,9,10], along with vaccine development [11]. Although several commercialized drugs, such as Remdesivir [12] or Hydroxychloroquine [13], were initially repositioned as antivirals to treat severe COVID-19, subsequent large randomized clinical trials showed them to be ineffective or to exhibit unacceptable levels of side effects [14,15,16]. More recently, an effective oral inhibitor of the SARS-CoV-2 main protease, M^pro^, Nirmatrelvir [17], administered in combination with Ritonavir for improved pharmacokinetics, was shown to exert antiviral activity and to prevent progression of COVID-19 to a more severe disease [18,19].

The SARS-CoV-2 main protease, known as 3CL^pro^ or M^pro^, exerts an essential role in viral replication [20,21], and inhibition of the conserved SARS-CoV M^pro^ results in effective infection control in cultured cells [21,22]. Furthermore, the substrate specificity of M^pro^ enzymes from SARS-CoV-2, SARS-CoV, MERS-CoV, enteroviruses, rhinoviruses, and noroviruses cleave the peptide bond following Gln on Leu-Gln↓[Ser, Ala, Gly], a cleavage specificity unknown in mammalian enzymes [21,23]. As a corollary, M^pro^-specific inhibitors are predicted to have negligible off-target activity and thus limited side effects [24,25]. These features make M^pro^ an attractive and obvious target to treat COVID-19. Designed drug-like compounds such as noncovalent inhibitors [26], peptidomimetic inhibitors [23], drug repositioning and screening of natural compounds [5,6,7,8,9,10,21] are some examples of the strategies used to discover small molecules targeting SARS-CoV-2 M^pro^. In this regard, Virtual Screening represents an effective and reliable computational approach useful for the rapid identification of bioactive compounds directed against a target of interest [27,28], and thus many in silico studies have applied Virtual Screening protocols as the initial step towards the identification of potential M^pro^ inhibitors [29,30,31,32,33], some of which have been experimentally confirmed [34,35,36,37].

Among the existent sources of compounds and databases, natural products stand out as one of the most interesting for drug discovery because of their large structural diversity and generally good bioavailability [8,32,33,34,35,36,37,38,39]. The use of natural compounds present in medicinal herbs has been part of popular knowledge in all cultures [34,40,41,42], including the use of natural anti-inflammatories or antivirals as therapeutic alternatives with at least some evidence of clinical efficacy [43,44,45,46]. In this context, Peruvian medicinal plants are traditionally known to be effective against many different respiratory diseases [41,42,46]. However, although some computational studies have been performed [47], no experimental studies have been reported to date exploring natural compounds from the autochthonous Peruvian flora for the treatment of COVID-19. Motivated by this knowledge gap, we have herein undertaken the identification of natural products targeting the SARS-CoV-2 M^pro^ main protease dimer, with a focus on the autochthonous flora of Peru as a source of bioactive compounds. To this end, we first performed an extensive literature search to identify and select compounds with known inhibitory activity against related viruses as candidate molecules. Subsequently, ensemble Molecular Docking of the selected compounds was performed for six different conformation representatives of the protease dimer, identified by conventional (cMD) and Gaussian accelerated Molecular Dynamics (GaMD). Candidates exhibiting the best complex binding free energies were subjected to extensive cMD simulations. Through the iterative evaluation of the free binding energy profiles, compounds exhibiting the best energetic behaviors were selected and experimentally tested in M^pro^ activity assays in vitro. This workflow has yielded Hyperoside, found in the autochthonous Peruvian plant *Chamaesyce thymifolia*, as an in silico predicted and experimentally confirmed inhibitor of SARS-CoV-2 M^pro^.

## 2. Results

### 2.1. Selection of Natural Compounds from Peruvian Flora

**Figure 1:**
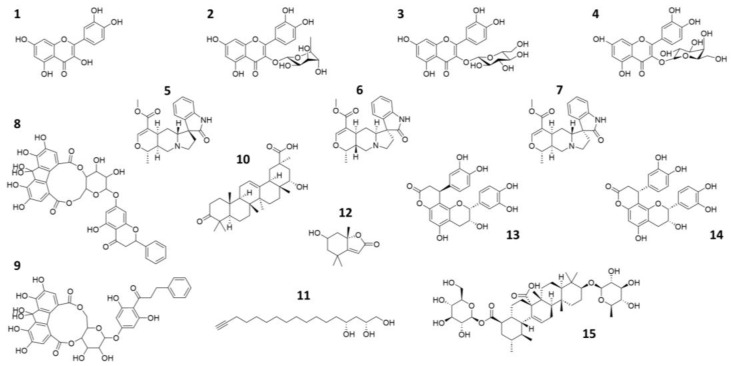
2D representation of the natural compounds selected for the present in silico study. Quercetin (**1**), Quercitrin (**2**), Isoquercetin (**3**), Hyperoside (**4**), Speciophylline (**5**), Mitraphylline (**6**), Uncarine—F (**7**), oligophenolic compound SCH 644343 (**8**), oligophenolic compound SCH 644342 (**9**), 22α-hydroxy-12-en-3-oxo-29-oic acid (**10**), (2R,4R)-1,2,4-trihydroxyheptadec-16-yne (**11**), (6S,7aR)-6-hydroxy-4,4,7a-trimethyl-6,7-dihydro-5H-1-benzofuran-2-one (**12**), Cinchonain Ia (**13**), Cinchonain Ib (**14**) and (28)-b-D-glucopyranosyl ester (**15**).

We conducted an extensive literature search based on bioactive natural compounds present in the Peruvian autochthonous flora. As a result, we initially identified up to 20 compounds exhibiting antiviral activity against related viruses as candidate inhibitors of SARS-CoV-2 M^pro^. Of these, 15 compounds were finally selected for subsequent in silico and in vitro studies, based on commercial availability (Table 1 and Figure 1). Since Quercetin exhibited inhibitory activity against M^pro^ through experimental screening [10,48], we finally decided not to include the compound in this study.

### 2.2. Representative Structures of the SARS-CoV-2 M^pro^ Protease Dimer

**Figure 2:**
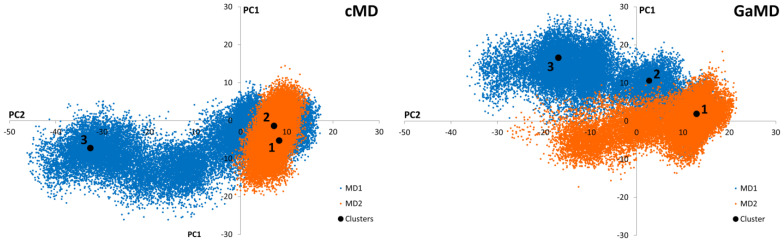
Graphical representation of the first two Principal Components identified from the cMD and GaMD simulations. Black dots correspond to the positions of the cluster representatives presenting a population higher than 10% of the total. Three representatives from each cluster were selected for each cMD and GaMD. Orange and blue colors represent the different simulation replicates.

Conventional Molecular Dynamics (cMD) and Gaussian-accelerated Molecular Dynamics (GaMD) simulations were applied to SARS-CoV-2 M^pro^ in order to identify independently clustered conformations and their representatives. The conformational diversity of the M^pro^ dimer binding site was depicted by means of an iterative process based on Root-Mean Square Fluctuation (RMSF) calculations (Supporting Figure S1). More specifically, amino acids presenting small fluctuations at the binding site during the simulation were used for Cα superposition of the structures in subsequent Root-Mean Square Deviation (RMSD) calculations (Supporting Figure S2). Further, binding site residues exhibiting larger RMSF values were considered to calculate the distance (RMSD) for the clustering process. Hence, residues with the lowest values from the RMSF analysis of the binding site amino acids were selected for the superposition of the structures, while those with the highest values were employed for PCA of the covariance matrix (Supporting Figure S3). Subsequently, three representatives from both cMD and GaMD simulation runs were selected, considering clusters with a population larger than 10% (Figure 2). Thus, a total of six receptor structures were employed in the virtual screening process, as most representative of the diverse conformational space of the M^pro^ dimer accessible for ligand binding (Supporting Figure S4).

### 2.3. Virtual Screening

Ensemble Molecular Docking of the selected natural compounds was performed on the 6 M^pro^ representatives, by applying the standard protocol of the QuickVina2 software (release 2015; Glossary, A. et al.) [81]. For each of the six M^pro^ representatives, 15 poses were generated per ligand, thus yielding a total of 1260 complexes to evaluate. To reduce the number of conformations for further study, only the poses exhibiting the best binding affinities for each representative were selected. Based on the PCA results obtained with the most populated cMD representative and prior studies by us [8], a threshold of −7.0 kcal/mol was established for the initial selection process of the best candidates. Consequently, only ligand-protease complexes exhibiting a scoring function value smaller than the threshold were selected for further analysis. Hereafter, selected complexes were solvated in explicit TIP3P water molecules [82] and subjected to a minimization protocol to allow free movement of the system. Then, the free binding energies of the structures were computed using Molecular Mechanics Poisson-Boltzmann Surface Area (MMPBSA) [83] and Molecular Mechanics Generalized Born Surface Area (MMGBSA) [84]. Finally, after ranking the complexes selected from the initial docking process, a second selection prioritizing the best free binding energies was sufficient to reduce the number of poses to be further evaluated in subsequent steps.

At the end of this process, the top-ranked 9 ligands were selected, each of them presenting up to 8 poses with each of the six M^pro^ conformational representatives. As a result, compounds Hyperoside, Isoquercetin, the oligophenolic compounds SCH 644343 and 644342, Quercitrin, Quinovic acid Glycoside, Cinchonain Ia, Cinchonain Ib and Loliolide, were selected to continue with the virtual screening process, while THHY, 22α-hydroxy-12-en-3-oxo-29-oic acid, Speciophylline, Mitraphylline and Uncarine-F were discarded. The selected compounds were heated to 300 K and density-equilibrated to be subjected to 100 ns cMD simulations. After simulations of the selected complexes, the time evolution of the thermal MMGBSA free binding energy was computed for all of them. By analyzing the energetic profiles and the binding energy averages of the last step of the simulation (Supporting Table S1), only the best complex poses exhibiting the best energetic profiles were selected for further analysis. Hence, up to three best poses were selected by compound and subjected to the extension of the simulations, except for Loliolide, which was discarded due its small binding energies with all the M^pro^ representatives. 

Repeating the procedure, extending the simulations up to 500 ns, 1 µs, 1.5 µs and 2 µs, and calculating the time evolution of the MMGBSA free binding energy of the selected complexes at each step (Supporting Tables S2–S5), we identified Hyperoside, the oligophenolic compounds SCH 644343, SCH 644342, Cinchonain Ia and Cinchonain Ib as the best candidates for experimental in vitro testing (Supporting Figure S5).

### 2.4. In Vitro Assays

Due to the commercial unavailability of the oligophenolic compounds SCH 644343 and SCH 644342 at the time of performing the experimental analysis of the selected compounds, the in vitro assays were limited to Hyperoside, Cinchonain Ia and Cinchonain Ib. The activity of these compounds against the SARS-CoV-2 M^pro^ main protease dimer, evaluated by means of a continuous assay employing a peptide FRET substrate (see Section 4.6.2), showed that, of the 3 compounds tested, only Hyperoside exhibited a specific inhibitory activity with a *K*_i_*^app^* (apparent inhibition constant) of 76 µM (Supporting Figure S6). In contrast, Cinchonain Ia Cinchonain Ib did not exhibit inhibitory activities at substrate concentrations below 125 µM, possibly as a reflection of physicochemical properties of the tested compounds under the experimental conditions employed that may have resulted in failure to bind to the receptor (Table 2). If Hyperoside were a competitive inhibitor, its intrinsic inhibition constant would be 27 μM, according to Equation (8). However, Hyperoside seems to act allosterically on M^pro^, and the intrinsic inhibition constant should be calculated using Equation (11). Because the conformational equilibrium constant *K* is not known, it is not possible to calculate a precise value for *K_i_*. However, because *K* must be larger than 1 (i.e., the active substrate-binding state must predominate over the inactive inhibitor-binding state), the *K_i_* should be much lower than 20 μΜ (calculated for *K* = 1), a considerable inhibitory potency for Hyperoside.

### 2.5. Binding Analysis

**Figure 3:**
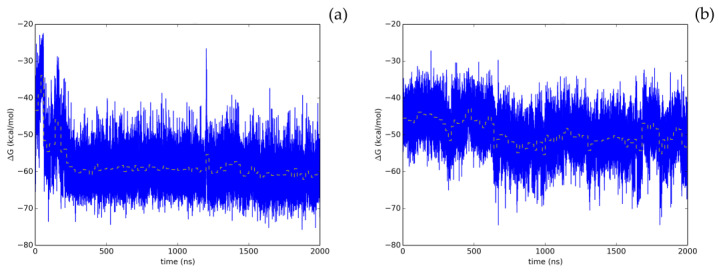
Time evolution of the thermal MMGBSA binding free energy obtained from the full length extended molecular dynamic simulations for the two allosteric binding sites identified for Hyperoside. Energetics profiles shown correspond to the first (**a**) and second (**b**) binding poses exhibiting the best energetic profiles, with computed average binding energies during the last 20 ns of −60.1 kcal/mol and −53.3 kcal/mol, respectively. Average binding energies computed every 20 ns of the simulation are depicted in the graphs as a yellow dashed line.

Therefore, the screening protocol followed in this study, based on our prior work [8], has permitted to accurately predict a natural compound with a smooth energetic profile (Figure 3), Hyperoside, as a small molecule displaying a significant enzymatic inhibitory activity on SARS-CoV-2 M^pro^.

**Figure 4:**
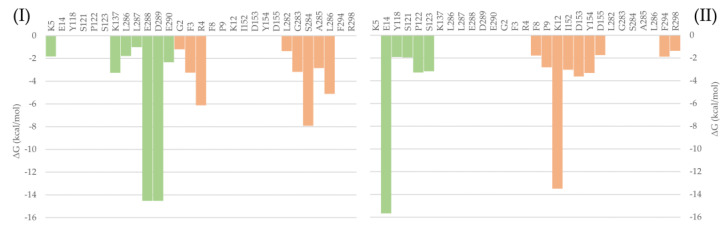
Free binding energy per residue decomposition for the two allosteric binding sites of Hyperoside in complex with the SARS-CoV-2 M^pro^ main protease dimer. On the left (**I**), the best binding site identified, with a free binding energy of −60.1 kcal/mol. On the right (**II**), the second binding site identified, with a free binding energy of −53.3 kcal/mol. The different monomers of the dimer are depicted in light green (Chain A) and light orange (Chain B).

**Figure 5:**
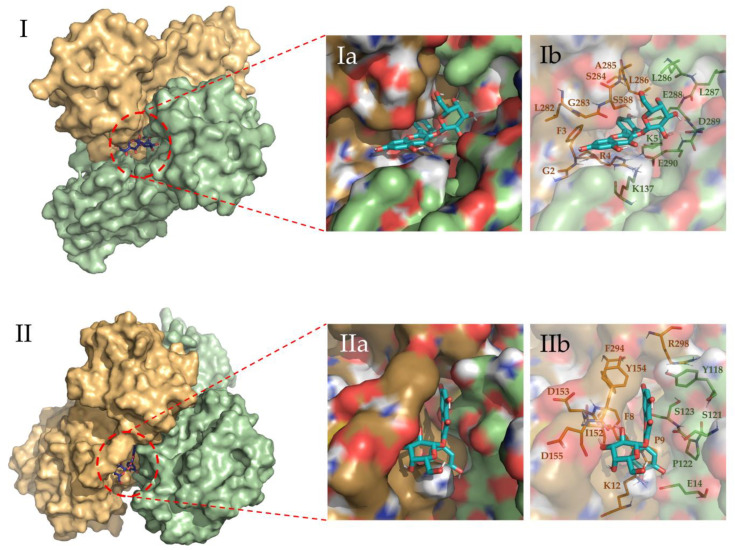
Binding site representation of the selected complexes for Hyperoside, at the last snapshot of their 2 µs molecular dynamic simulations. The two monomers of the M^pro^ SARS-CoV-2 main protease dimer are depicted in light green (Chain A) and light orange (Chain B). Allosteric interactions depicted correspond to the first (**I**) and second (**II**) most stable binding sites identified for Hyperoside, with binding energies of −60.1 kcal/mol and −53.3 kcal/mol, respectively. (**Ia**) and (**Ib**) representations show the ligand bound to the ligand pocket, and (**IIa**) and (**IIb**) representations depict the spatial distribution of the residues defining the interacting pocket.

To evaluate the most relevant interactions between Hyperoside and residues on the M^pro^ dimer, a free energy decomposition analysis of the last 100 ns of simulation was performed for the two allosteric sites identified (Figure 4, Supporting Table S6). It is important to note that our initial docking was performed on the described binding site of the M^pro^ protein. However, because of molecular dynamics simulations, the ligand was found to relocate to the final allosteric sites. Visual inspection of the binding mode of the poses evaluated afforded the inference of different interaction profiles of Hyperoside with two distinct regions of the dimer interphase (Figure 5(Ib,IIb)). As such, we found that interactions between Hyperoside and E288 and D299 of the protease dimer chain A, and S284 of the protease dimer chain B, contributed the most to the complex formation at the first binding site evaluated (−60.1 kcal/mol), with contributions of −14.5 kcal/mol, −14.5 kcal/mol and −7.9 kcal/mol, respectively. For the second binding site (−53.3 kcal/mol), interactions between Hyperoside and E14 (chain A) and K12 (chain B) contributed with −15.7 kcal/mol and −13.5 kcal/mol, respectively (Supporting Table S6). 

**Figure 6:**
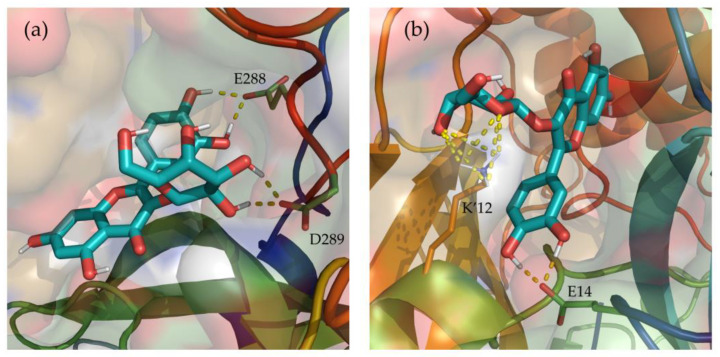
Depiction of the most relevant hydrogen bonds established by Hyperoside, and the two allosteric binding sites identified, for the last 100 ns of their 2 µs molecular dynamic simulations. Representations correspond to the first (**a**) and second (**b**) most stable binding sites identified for Hyperoside. Ligand—M^pro^ hydrogen bonds are represented as a yellow dashed line. The two monomers of the SARS-CoV-2 M^pro^ dimer are depicted in light green (Chain A) and light orange (Chain B). Only the lateral chains of the residues presenting this type of interaction are shown.

Additionally, hydrogen bonds established between Hyperoside and the residues of the two binding sites identified were evaluated during the last 100 ns of simulation (Supporting Table S7). From the analysis, hydrogen bonds with occupancies greater than 90% of the simulation correspond to acceptor-donor interactions with residues E288 and D289, and the hydroxyl groups of the ligand at the first binding site (Figure 6a). For the second binding site, acceptor-donor interactions between residue E14 and the hydroxyl groups of the ligand were identified as the major contributor, as well as the hydrogen bonds established by the oxygen atoms of the ligand and residue K12 from chain B of the protease dimer (Figure 6b).

Through visualization of the binding poses for the selected compounds obtained at the end of the 2 µs simulations, two different allosteric binding sites in the dimer interphase region were identified for Hyperoside, as depicted in Figure 5. For the oligophenolic compounds SCH 644343 and SCH 644342, allosteric binding sites were also identified computationally. In the case of Cinchonain Ia and Cinchonain Ib, allosteric binding sites were identified as stable for each of the compounds, despite exhibiting converged energetic profiles for the active center in poses that were finally discarded during the evaluation process.

Despite the relative stability of Cinchonain Ia and Cinchonain Ib, the experimental assays showed no inhibitory activity of these compounds on the protease dimer. In addition to potential physicochemical issues pertinent to the assay experimental conditions, it is possible that binding energy fluctuations found during the evaluation of the full trajectory may have contributed to the lack of inhibitory activity of these compounds (Supporting Figure S5e,f). Regarding the energetic profile of the binding sites identified for Hyperoside, it is evident that the first allosteric binding site (−60.1 kcal/mol) presents a stable energetic profile during the last 1750 ns of the simulation. This binding site, defined by residues K5, K137, L286, L287, E288, D289, E290 from chain A and G2, F3, R4, L282, G283, S284, A285 and L286 from chain B, corresponds to the allosteric binding site previously identified through fragment screening as a promising opportunity for allosteric modulation of the SARS-CoV-2 M^pro^ dimer [85]. In addition, the second allosteric binding site (−53.3 kcal/mol) defined by residues E14, Y118, S121, P122 and S123 from chain A and F8, P9, K12, L152, D153, Y154, D155, F294 and R298 from chain B, exhibited a good energetic profile despite the small fluctuations presented during the complete simulation (Figure 3b, Supporting Table S6).

### 2.6. Evaluation of ADME Properties

The safety and efficacy of drug compounds in the drug discovery process depend on their drug-likeness and ADME properties. In general, poor solubility and low membrane permeability reduce their cell activity. Predicted values for these properties are reported in Tables S9 and S10. From a medicinal chemistry standpoint, Hyperoside has a moderate synthetic accessibility score (5.32) on a scale of 1 to 10, where 1 indicates very easy and 10 indicates very difficult. The compound is expected to be water-soluble and have a greater affinity for water than for oil. Moreover, experimental studies show Hyperoside to be highly soluble and to exhibit preference for water [86]. However, Hyperoside violates two of Lipinski’s rules, which suggests the possibility that it may not be active/absorbable orally. Additionally, low gastrointestinal absorption is predicted. Despite these possible problems, multiple and diverse studies are being carried out to see its possible use in the treatment of many diseases [87].

## 3. Discussion

In response to the urgent need to identify effective treatments for COVID-19, many in silico and in vitro studies based on repositioning and natural products have been conducted. In the present work, we selected a set of 15 bioactive natural compounds from the autochthonous Peruvian flora exhibiting antiviral activity against related viruses and, analyzed them through a comprehensive in silico approach based on the energetics of the complexes formed between the compounds and 6 representative confirmations of the SARS-CoV-2 main protease dimer, M^pro^.

After applying our multistep Virtual Screening pipeline, five out of the initial natural compounds were singled out as potential SARS-CoV-2 M^pro^ main protease inhibitors. Three of the compounds were experimentally tested in in vitro assays with recombinant M^pro^, of which one, Hyperoside, displayed significant inhibitory activity, likely through an allosteric modulation mechanism.

Given the promising results found for Hyperoside, the allosteric interactions identified for the compound were evaluated through a free binding energy per residue decomposition analysis, as a means to identify the amino acids involved in the formation of the complexes, as well as by visual inspection of the binding modes. This analysis highlights residues K5, K137, L286, L287, E288, D289, E290 (chain A) and G2, F3, R4, L282, G283, S284, A285, L286 (chain B); and E14, Y118, S121, P122, S123 (chain A) and F8, P9, K12, L152, D153, Y154, D155, F294, R298 (chain B), involved in the two different allosteric interactions identified for Hyperoside, as playing an important role in its allosteric modulation of the activity of the SARS-CoV-2 M^pro^ dimer.

Additionally, based on the promising in silico results obtained for the oligophenolic compounds SCH 644343 and SCH 644342, not tested experimentally due to their commercial unavailability, we suggest them as possible candidates for future in vitro studies to target the SARS-CoV-2 M^pro^ main protease dimer.

A general corollary to our results is that the protocol followed has been effective to accurately identify at least one natural inhibitor of the SARS-CoV-2 M^pro^ enzymatic activity exhibiting a converged energetic profile, reinforcing prior efforts by us [8], thus demonstrating the effectiveness of the virtual approach, which allows successful predictions of the best candidate compounds to be tested experimentally as allosteric inhibitors of target enzymes.

As a final remark, we must note that drug-likeness and ADMET properties of compounds govern their safety and efficacy. In general, cell activity would be further reduced due to solubility and membrane permeability issues. For this reason, these properties were evaluated for Hyperoside using the SwissADME web tool.

## 4. Materials and Methods

### 4.1. Selection of Bioactive Compounds with Antiviral Activity Characterized from Peruvian Medicinal Herbs

#### 4.1.1. Literature Search for Bioactive Peruvian Natural Compounds

A literature search was conducted through PubMed and Google Academic towards the identification of antiviral compounds characterized from Peruvian herbs and reported in the literature. The following keywords were used for the search: “Peruvian medicinal plants AND bioactive compounds AND antiviral activity”. Both primary and secondary references including original papers, reviews, books, systematic reviews and meta-analyses were selected for review. Publications in English and Spanish were included. Both grey and incomplete records were excluded.

The authors reviewed the full-text papers and obtained relevant data, including the plant’s scientific name, common name, paper title, bioactive compound(s) characterized from that species, SMILE, bioactivity, virus name, extraction technique and bioassay. SMILES used for this study can be found in Supporting Table S8.

The selected natural compounds were identified from original papers where those molecules were extracted and characterized from Peruvian autochthonous plant species and where antiviral activity was demonstrated by in vitro and in vivo assays.

#### 4.1.2. Preparation of the Selected Natural Compounds

Each selected natural compound structure was initially prepared from the simplified molecular-input line-entry system (SMILES) ASCII string using the Maestro 2016-2 software (release 2016; Schrödinger LLC, New York, NY, USA) [88]. Ligand charges and parameters were obtained using Antechamber from AMBER20 [89]. The generalized Amber forcefield (GAFF2) [90] was used for parameter obtention, whereas the RESP method [91] was employed for the computation of partial charges.

Protonation and energy minimization for all compounds were performed with Maestro 2016-2 [88]. Finally, a database with the studied compounds was generated for the subsequent calculations.

### 4.2. Selection of Representative Structures for the SARS-CoV-2 M^pro^ Protease Dimer

#### 4.2.1. Preparation of the System

To represent the structural diversity of the target protein, the dimeric crystallographic structure of SARS-CoV-2 M^pro^ protease, with PDB access code 6Y84, was selected as the initial structure employed in the present work. Using the LigPrep module included in Maestro 2016-2 [88], allowed missing hydrogens to be added, given their protonation state at pH 7.0, and side chains orientations were set up. The protein was then dissolved with TIP3P water molecules [82] in a cubic simulation box whose dimensions were determined by fixing a minimum distance of 15 Å between the box walls and the solute. To avoid bad contacts, water molecules nearer than 1.2 Å to any protein atom were removed. To neutralize the system, four Na^+^ ions were added at the positions of lowest electrostatic potential using the AMBER20 Leap module [89]. The ff19SB force field [92] was applied for all calculations, using a cut-off of 10 Å for noncovalent interactions. The PME method [93] was used to treat electrostatic interactions.

#### 4.2.2. Energy Minimization

Prior to any MD simulation, the structure was initially relaxed in a 5000-step procedure using the steepest descent minimization method to eliminate possible steric clashes. First, relaxation was only allowed for ions and water molecules, keeping all the protein atoms fixed by applying harmonic positional constraints of 5 kcal/mol·Å^−2^. Only the main atoms of the protein were retained in a second step. Last, in a third step, all the atoms in the system were allowed to move.

#### 4.2.3. Molecular Dynamics Simulations

After minimization, the system was heated to 300 K in 30 K intervals every 20 ps under the NVT ensemble. The heating process was performed by maintaining the positions of the main protein atoms fixed through the imposition of the abovementioned harmonic positional constraint, using the Langevin thermostat algorithm and a collision frequency of 3 ps^−1^. Therefore, 2 ns of density equilibration protocol was conducted under the NPT ensemble, maintaining fixed the main atoms of the protein with harmonic positional restrictions of 0.1 kcal/mol·Å^−2^. Then, to increase the exploration of the conformational space of the system, conventional molecular dynamics (cMD) and Gaussian accelerated molecular dynamics (GaMD) of 500 ns length were run by duplicated under the NVT ensemble [94]. After applying a density equilibration protocol, an intermediate step of 20 ns was performed for the GaMD simulations to obtain the initial statistical analysis of the applied dual boost potential. For this step, the standard deviation upper limit of the total potential boost (σ_0P_) and the dihedral potential boost (σ_0V_) were set to 3 and 5, respectively. Simulations were carried out using a cut-off of 11 Å applying a switch function at 8 Å. All the trajectories and previous minimization protocols were computed using the PMEMD (Particle Mesh Ewald Molecular Dynamics) code of the AMBER20 software [89] in its CUDA version using the AMBER ff19SB force field [92].

#### 4.2.4. Root-Mean Square Deviation (RMSD) and Root-Mean Square Fluctuation (RMSF) Analysis

To evaluate the system’s structural stability during the MD simulations, Root-Mean Square Deviation (RMSD) for all the trajectories were computed using the Cpptraj module from AMBER20 [89,95] using as reference the last minimized structure. To select the alpha carbons (Cα) with lowest fluctuations, an iterative procedure in which all residue Cα were used to reorient the structures in the first stage was employed. The Root-Mean Square Fluctuation (RMSF) of the superposed trajectories was then computed for all protein residues with Cpptraj. Residues with RMSF values smaller than the threshold were successively used in subsequent RMSD calculations. All Cα atoms were used in the superposition for the initial step. Then, cut-offs of 2.0, 1.0 and 0.5 Å were used in the selection of the atoms to be superposed in the subsequent second and third steps, respectively, with a total of 137 amino acids meeting the requirement. As a result, we obtained information related to the local conformational flexibility of the residues not superposed through the identification of the residues with lowest fluctuations during the MD simulation.

#### 4.2.5. Cluster Analysis

To represent the maximum structural diversity of the M^pro^ protease dimer active site, the average linkage algorithm [96] implemented in the Cpptraj module of AMBER20 [89,95] was used. For both cMD and GaMD simulations, MD simulation structures were grouped by similarity into 15 clusters, using as distance the RMSD of the Cα located in the binding site with a larger RMSF. A total of 86 amino acids were selected.

#### 4.2.6. Principal Component Analysis (PCA)

Principal Component Analysis (PCA) was used to identify the conformational space explored in the different simulations and to determine the differences between the representatives obtained after the clustering process. Furthermore, PCA is commonly employed in the dimension reduction needed to depict protein motions from the lowest to the highest contributions. To do so, a covariance matrix was built considering all the different conformations identified in the simulations, using the Cα coordinates of the residues used for the clustering process. To obtain the Principal Components, the covariance matrix was diagonalized to obtain the eigenvectors (PC(i), i = 1, N), with N corresponding to the number of previously selected protein residues, and associated eigenvalues λ(i). Once the eigenvalues were ordered from the highest to the lowest contribution, the first components defining the most important protein motions were identified [97]. PCA calculations were performed using the Cpptraj module of AMBER20 [89,95].

### 4.3. Virtual Screening for Ligands of the SARS-CoV-2 M^pro^ Protease Dimer

**Figure 7:**
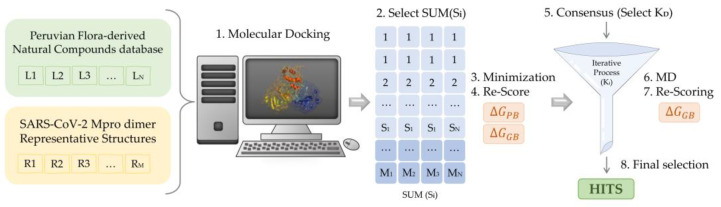
Scheme of the multistep virtual screening procedure carried out in the present work.

For each of the M^pro^ representatives, a multistep Virtual Screening based on an initial docking of the selected Peruvian flora-derived natural products was performed (Figure 7). Firstly, the AutoDock QVina2 software [81] was used to dock the selected compounds. A target-based docking process was conducted by the definition of the coordinate grid box of dimensions 37.5 × 45.0 × 41.25 Å^3^, placed at the active site defined by C145, L27 and H41 residues. Then, for each M^pro^ representative, a free binding energy threshold of −7.0 kcal/mol was established for the selection of the best protein-ligand complexes. Further analysis was performed for the selected docking poses. Ligands were parametrized with the generalized amber force field gaff2 [90] and the ff19SB forcefield [92] for the target protein representatives, using the Antechamber module of AMBER20 [89]. Leap module was employed in the simulation box construction and complex solvation with TIP3P water molecules [92]. Neutralization of the system was ensured by the addition of counterions. Once prepared, each solvated complex simulation box was relaxed following a multistep minimization process, as previously described by us [8]. Briefly, to eliminate undesired steric clashes, an initial minimization process was performed exclusively for solvent and counterion molecules, followed by a second minimization run allowing the free movement of the ligand. Finally, free movement was enabled for all atoms. Thereafter, free binding energy was computed for all the minimized complexes using Molecular Mechanic Poisson-Boltzmann Surface Area (MMPBSA) [83] and Molecular Mechanics Generalized Born Surface Area (MMGBSA) [84]. Once obtained, a scoring of the ligands was conducted to rank order the evaluated complexes. Using an energy criterion, only the best complexes were selected for further MD simulations analysis, in which MMGBSA free binding energies of the complete trajectory were evaluated. Hence, the ligands exhibiting the best energies and converged behavior were iteratively selected for extension and subsequent free binding energy recalculation of their MD simulations. The final selection was based on the best and smoothest energetic behavior of the most extended simulations. Then, an interaction analysis of the ligand at the binding site was performed.

### 4.4. Free Binding Energy Calculation

Free binding energies were calculated using the MMPBSA and the MMGBSA procedures [83,84] implemented in AMBER20 [89]. For both MMPBSA and MMGBSA approaches, the same expression is used in the computation of the free binding energy [98]. Thus, the free energy for binding of the ligand (L) to the protein receptor (R) to form the complex (RL),

(1) $${∆G}_{\mathrm{binding}\;}=G_{\mathrm{RL}\;}-G_{L\;}-G_{R\;}$$

can be decomposes into contributions of different interactions as

(2) $${∆G}_{\mathrm{binding}\;}={∆E}^{\mathrm{MM}}+{\;∆G}^{\mathrm{solv}}-\;T{∆S\;}^{}$$

where ${∆H}^{\mathrm{MM}}$ corresponds to the gas-phase interaction energy obtained by the summation of the internal energy, the noncovalent van der Waals ($\Delta E_{\mathrm{vdW}}^{}$) and electrostatic ($\Delta E_{\mathrm{elec}}^{}$) molecular mechanics energies. ∆G^solv^; however, corresponds to the solvation free energy, calculated as the addition of the polar ($\Delta G_{\mathrm{polar}}^{\mathrm{solv}}$) and non-polar terms ($\Delta G_{\mathrm{nonpolar}}^{\mathrm{solv}}$). $\Delta G_{\mathrm{polar}}^{\mathrm{solv}}$ is determined numerically by solving the Poisson-Boltzmann (PB) equation [99] or the Generalized Born (GB) [100], the simplified form for MMPBSA and MMGBSA algorithms, respectively. The Onufriev-Bashford-Case (OBC) generalized Born method (igb = 2) [101] was employed for the calculations. Hereafter, the $\Delta G_{\mathrm{nonpolar}}^{\mathrm{solv}}$ term is calculated as follows:

(3) $${∆G}_{\mathrm{nonpolar}\;}^{\mathrm{solv}}=\gamma \;\mathrm{SASA}+\beta \;$$

where the Solvent-Accessible Surface Area (SASA) was computed through the LCPO approach [102], respectively, setting γ and β constants to 0.00542 kcal/mol·Å^2^ and 0.92 kcal/mol for MMPBSA [83] and 0.0072 kcal/mol·Å^2^ and 0 kcal/mol for MMGBSA [84]. All calculations were performed using the MMPBSA.py python program [103].

### 4.5. ADMET Evaluation of the Active Compounds

Given the significance of assessing the absorption, distribution, metabolism, excretion, and toxicity (ADMET) properties of any potential drug candidate, we used the SwissADME module [104], available on the Swiss Institute of Bioinformatics (SIB) webserver (https://www.sib.swiss (accessed on 5 April 2023)) to evaluate the ADME profile of the active compounds. Properties relevant to medicinal chemistry, drug-likeness, pharmacokinetics, hydrophilicity, and Lipophilicity have been computed. Results can be found at Supporting Tables S9 and S10.

### 4.6. In Vitro Assays for the Selected Compounds

#### 4.6.1. SARS-CoV-2 M^pro^ Expression and Purification

SARS-CoV-2 M^pro^ was expressed and purified as reported previously [48,105,106]. Briefly, transformed BL21 (DE3) Gold *E. coli* were grown with LB/ampicillin (100 μg/mL) at 37 °C overnight and protein expression was induced with 1 mM isopropyl 1-thio-β-D-galactopyranoside (IPTG) at 18 °C for 5 h. Harvested cells were resuspended in lysis buffer (sodium phosphate 50 mM, pH 7, sodium chloride 500 mM). After sonication on ice, cells were treated with benzonase (20 U/mL) and lysozyme (0.5 mg/mL). Soluble protein was obtained by centrifugation and filtration. His-tagged protein was purified in a single step by immobilized metal affinity chromatography applying a 10–250 mM imidazole gradient. Pooled fractions were dialyzed in buffer sodium phosphate 50 mM, pH 7, sodium chloride 150 mM. An extinction coefficient of 32,890 M^−1^ cm^−1^ at 280 nm was used for determining M^pro^ concentration.

#### 4.6.2. SARS-CoV-2 M^pro^ Proteolytic Activity Assay

The substrate (Dabcyl)KTSAVLQSGFRKME(Edans)-NH2 (Biosyntan GmbH), labelled with a Förster resonance energy transfer (FRET) donor-acceptor couple, was employed in vitro to monitor M^pro^ activity. A final concentration of 20 μM substrate was added to a final concentration of 0.2 μM enzyme in a final volume of 100 μL, in buffer sodium phosphate 50 mM, pH 7, NaCl 150 mM. Fluorescence emission (λ_excitation_ = 380 nm, λ_emission_ = 500 nm) was continuously measured in a FluoDia T70 microplate reader (Photon Technology International). Enzymatic activity was quantified as the initial slope of the fluorescence emission vs. time traces. Kinetic parameters, the Michaelis-Menten constant *K_m_* and the catalytic rate constant *k_cat_*, were previously estimated for M^pro^ (*K_m_* = 11 μM and *k_cat_* = 0.040 s^–1^).

#### 4.6.3. SARS-CoV-2 M^pro^ Inhibition Assay

The inhibition potency of compounds was assessed by determining the inhibition constant, *K_i_*, and the half-maximal inhibitory concentration, IC50. Serial dilutions (2-fold) of compounds (from 125 μΜ to 0 μM) were added to 0.2 μM enzyme concentration and 20 μM substrate concentration. maintaining a constant DMSO percentage when required. M^pro^ activity (initial slope of the fluorescence emission traces) as a function of compound concentration, normalized by the activity (slope) in the absence of compound, provided the residual activity at a given compound concentration. A quasi-simple inhibition model allowed the estimation of the apparent inhibition constant, *K_i_^app^*, for each compound by non-linear regression analysis, according to the following scheme:

(4) $$\begin{array}{c} E+I↔EI\\ K_{i}^{app}=\frac{\left[E\right]\left[I\right]}{\left[EI\right]} \end{array}$$

where [*E*] is the free enzyme concentration, [*I*] is the free inhibitor concentration, and [*EI*] is the enzyme-inhibitor complex concentration. Solving the associated quadratic equation allowed the calculation of the free concentration of inhibitor for given values of the total enzyme and inhibitor concentrations, [*E*]_*T*_ and [*I*]_*T*_, and the inhibition curve was built by plotting ratio of the initial velocities, *v*([*I*])/*v*([*I*] = 0), as a function of total inhibitor concentration:

(5) $$\begin{array}{c} \frac{v\left(\left[I\right]\right)}{v\left(\left[I\right]=0\right)}=1-\frac{\left[EI\right]}{{\left[E\right]}_{T}}=\frac{1}{1+\frac{\left[I\right]}{K_{i}^{app}}}\\ \left[I\right]=\frac{1}{2}\left({\left[I\right]}_{T}-{\left[E\right]}_{T}-K_{i}^{app}+\sqrt{{\left({\left[I\right]}_{T}+{\left[E\right]}_{T}+K_{i}^{app}\right)}^{2}-4{{\left[E\right]}_{T}\left[I\right]}_{T}}\right) \end{array}$$

Along these calculations, inhibitor depletion (due to binding to enzyme) was considered; thus, no approximation for the free inhibitor concentration (e.g., assuming to be equal to the total inhibitor concentration) was made.

In the case the inhibitor acts through a purely competitive mechanism, the previous quasi-simple equilibrium can be expanded as a simple competitive inhibition model as follows (Equation (6)):

(6) $$\begin{array}{ccc} E+S & ↔ & \begin{array}{ccc} ES & \to & E+P \end{array}\\ + & & \\ \begin{array}{c} I\\ ↕\\ EI \end{array} & & \end{array}$$

from which the initial enzymatic velocity is expressed as:

(7) $$v\left(\left[I\right]\right)=k_{cat}{\left[E\right]}_{T}\frac{\left[S\right]}{K_{m}\left(1+\frac{\left[I\right]}{K_{i}}\right)+\left[S\right]}$$

where *k*_*cat*_ is the catalytic rate constant, *K*_*m*_ is the Michaelis-Menten constant, [*S*] is the substrate concentration, and *K*_*i*_ is the intrinsic (i.e., substrate concentration-independent) inhibition constant. From this, the inhibition curve can be written as:

(8) $$\begin{array}{c} \frac{v\left(\left[I\right]\right)}{v\left(\left[I\right]=0\right)}=\frac{1}{1+\frac{\left[I\right]}{K_{i}\left(1+\frac{\left[S\right]}{K_{m}}\right)}}=\frac{1}{1+\frac{\left[I\right]}{K_{i}^{app}}}\\ K_{i}^{app}=K_{i}\left(1+\frac{\left[S\right]}{K_{m}}\right) \end{array}$$

By approximating the free compound concentration by the total compound concentration and neglecting compound depletion, the *K_i_^app^* in the previous equation is equivalent to the IC50. It is important to emphasize that IC50 is not an appropriate inhibition potency index, because it is an assay-dependent parameter (it depends on [*E*]_*T*_, [*S*] and *K*_*m*_, and it is estimated without taking into consideration compound depletion).

If the inhibitor acts through an allosteric non-competitive mechanism, the previous quasi-simple equilibrium (Equation (6)) can be expanded as a simple allosteric inhibition model as follows (Equation (9)):

(9) $$\begin{array}{ccc} \begin{array}{ccc} E & + & S \end{array} & \begin{array}{cc} ↔ & ES \end{array} & \begin{array}{ccc} \to & E & \begin{array}{cc} + & P \end{array} \end{array}\\ \begin{array}{ccc} ↕ & & \end{array} & & \\ \begin{array}{ccc} E^{*} & + & I \end{array} & \begin{array}{cc} ↔ & E^{*}I \end{array} & \end{array}$$

from which the initial enzymatic velocity is expressed as:

(10) $$v\left(\left[I\right]\right)=k_{cat}{\left[E\right]}_{T}\frac{\left[S\right]}{K_{m}\left(1+\frac{1}{K}\left(1+\frac{\left[I\right]}{K_{i}}\right)\right)+\left[S\right]}$$

where *K* is the equilibrium constant for the conformational equilibrium between the inactive conformation E* stabilized by inhibitor binding and the active conformation *E* stabilized by substrate binding (*K* = [*E*]/[E*]), and, again, *K*_*i*_ is the intrinsic inhibition constant. From this, the inhibition curve can be written as:

(11) $$\begin{array}{c} \frac{v\left(\left[I\right]\right)}{v\left(\left[I\right]=0\right)}=\frac{1}{1+\frac{\left[I\right]}{K_{i}\left(1+K\left(1+\frac{\left[S\right]}{K_{m}}\right)\right)}}=\frac{1}{1+\frac{\left[I\right]}{K_{i}^{app}}}\\ K_{i}^{app}=K_{i}\left(1+K\left(1+\frac{\left[S\right]}{K_{m}}\right)\right) \end{array}$$

Once the apparent equilibrium constant *K_i_^app^* is estimated from the inhibition curve by non-linear least-squares regression analysis, the true inhibition constant *K*_*i*_ can be calculated using Equation (8) or Equation (11).

## Figures and Tables

**Table 1:** Bioactive compounds present in the Peruvian flora selected from the literature for the in-silico study. References for each of the selected compounds are indicated.

Scientific Name	Spanish Vernacular Name	Bioactive Compound
*Persea americana* [49,50]	Aguacate [49]	(2R,4R)-1,2,4-trihydroxyheptadec-16-yne (THHY) [51]
*Chamaesyce thymifolia* [50,52,53]	Cocodrilo o Golondrina [50,54]	Quercetin 3-O-b-glucoside (ISOQUERCETIN) [55,56,57]
		Quercetin 3-O-b-galactoside (HYPEROSIDE) [55,57,58,59,60,61,62]
*Maytenus macrocarpa* [50,63]	Chuchu washa [50]	22α-hydroxy-12-en-3-oxo-29-oic acid [64]
*Caesalpinia pulcherrima* [50,65,66,67]	Virundera del Perú	3,3′,4′,5,6-penta hydroxyflavone (QUERCETIN) [48,56,57,58,59,61,68,69,70,71]
		quercetin-3-rhamnoside (QUERCITRIN) [68,70,71]
*Stylogne cauliflora* [72]	Cauliflora	Oligophenolic Compound SCH 644343 [73]
		Oligophenolic Compound SCH 644342 [73]
*Phyllanthus urinaria* [50]	Chanca Piedra [50]	(6S,7aR)-6-hydroxy-4,4,7a-trimethyl-6,7-dihydro-5H-1-benzofuran-2-one (LOLIOLIDE) [74,75]
*Uncaria tormentosa* [76]	Uña de Gato	Speciophylline [74,77]
		Mitraphylline [74,77]
		Uncarine-F [74,77]
		(28)-b-D-glucopyranosyl ester (QUINOVIC ACID GLYCOSIDE) [78]
		Cinchonain Ia [79,80]
		Cinchonain Ib [79,80]

**Table 2:** M^pro^ inhibition values exhibited in in vitro assays by the compounds selected for experimental activity determinations. Compounds are ordered according to their best Free Binding Energies Computed for the complete Molecular Dynamics Simulations.

Compound	*K_i_* (µM)	IC_50_ (µM)	ΔG_biniding_ (MMGBSA) (kcal/mol) **
Hyperoside	27 (competitive) <20 (allosteric)	76	−60.1 −53.3
Cinchonain Ia	*	*	−59.0
Cinchonain Ib	*	*	−43.8

* Compounds with an asterisk correspond to those that have not exhibited detectable inhibitory activity at substrate concentrations below 125 µM. ** Two values are reported for Hyperoside, corresponding to the two binding sites identified.

## Data Availability

Data is contained within the article and Supplementary Materials.

## Supplementary Materials

The following supporting information can be downloaded at: https://www.mdpi.com/article/10.3390/ph16040585/s1, description. Figure S1. RMSF of each iteration step performed in the determination of the protein residues presenting the smallest RMSF values, for both cMD and GaMD simulations; Figure S2. RMSD time evolution for each of the four different steps used in the determination of the protein residues presenting the smallest RMSF values, for both cMD and GaMD simulations runs; Figure S3. SARS-CoV-2 M^pro^ protease dimer representation. Residues colored in blue and green respectively represent the regions used in the superimposition of the structures (low mobility), and in the clustering and PCA analysis (high mobility); Figure S4. Superposition of the six representative structures of the SARS-CoV-2 M^pro^ protease dimer, identified from cMD and GaMD simulations; Figure S5. Time evolution of the MMGBSA binding free energy obtained for the full length extended molecular dynamic simulations of 2000 ns for the selected compounds; Figure S6. In vitro SARS-CoV-2 M^pro^ protease dimer inhibitory activity assay of Hyperoside natural compound selected from the multistep in silico analysis performed; Table S1. Free binding energy computed with the MMGBSA approach for 100 ns of cMD simulation of the evaluated compounds in complex with the SARS-CoV-2 M^pro^ main protease dimer; Table S2. Free binding energy computed with the MMGBSA approach for 500 ns of cMD simulation of the evaluated compounds in complex with the SARS-CoV-2 M^pro^ main protease dimer; Table S3. Free binding energy computed with the MMGBSA approach for 1000 ns of cMD simulation of the evaluated compounds in complex with the SARS-CoV-2 M^pro^ main protease dimer; Table S4. Free binding energy computed with the MMGBSA approach for 1500 ns of cMD simulation of the evaluated compounds in complex with the SARS-CoV-2 M^pro^ main protease dimer; Table S5 Free binding energy computed with the MMGBSA approach for 2000 ns of cMD simulation of the evaluated compounds in complex with the SARS-CoV-2 M^pro^ main protease dimer; Table S6. Binding free energy decomposition by residue computed for the last 100 ns of the complete 2 µs of cMD simulation of the two selected poses for Hyperoside in complex with the SARS-CoV-2 M^pro^ main protease dimer; Table S7. Most important Hydrogen Bonds stablished during the last 100 ns of the complete 2 µs of cMD simulation of the two selected poses for Hyperoside in complex with the SARS-CoV-2 M^pro^ main protease dimer; Table S8. Isomeric SMILES of the bioactive compounds selected from the literature search; Table S9. Physicochemical properties of Hyperoside; Table S10. Medicinal chemistry, drug-likeness, pharmacokinetics, hydrophilicity, and lipophilicity properties of Hyperoside; File S1. File input for the QVina software; File S2. File to run CPPTRAJ to obtain the top 10 PCs for the full conventional MD trajectory.
