# Supplementary material for: Autochthonous Peruvian Natural Plants as Potential SARS-CoV-2 Mpro Main Protease Inhibitors
Source: Pharmaceuticals (Basel). 2023 Apr 13;16(4):585. doi: 10.3390/ph16040585 (PMC10146424; doi:10.3390/ph16040585)
Supplement: Supplementary file 1 [file pharmaceuticals-16-00585-s001.zip › pharmaceuticals-2289180-supplementary.pdf]

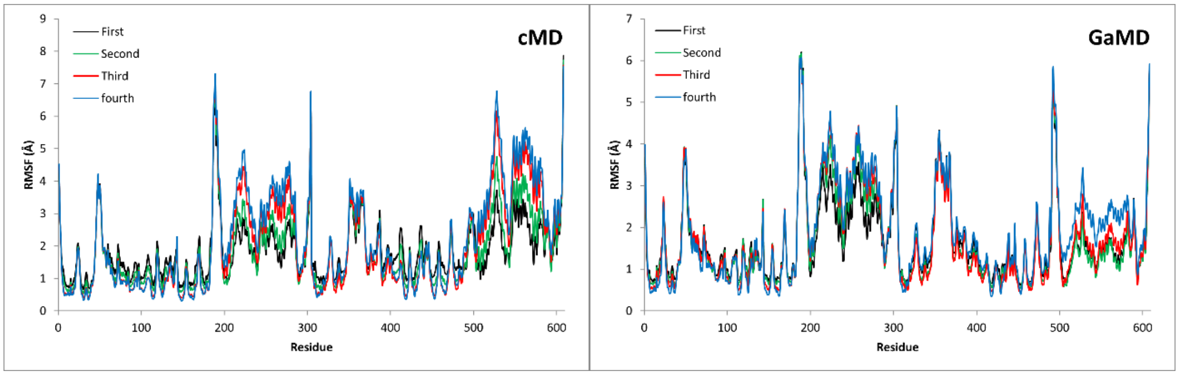

Figure S1. RMSF of each iteration step performed in the determination of the protein residues presenting the smallest RMSF values, for both cMD and GaMD simulations.

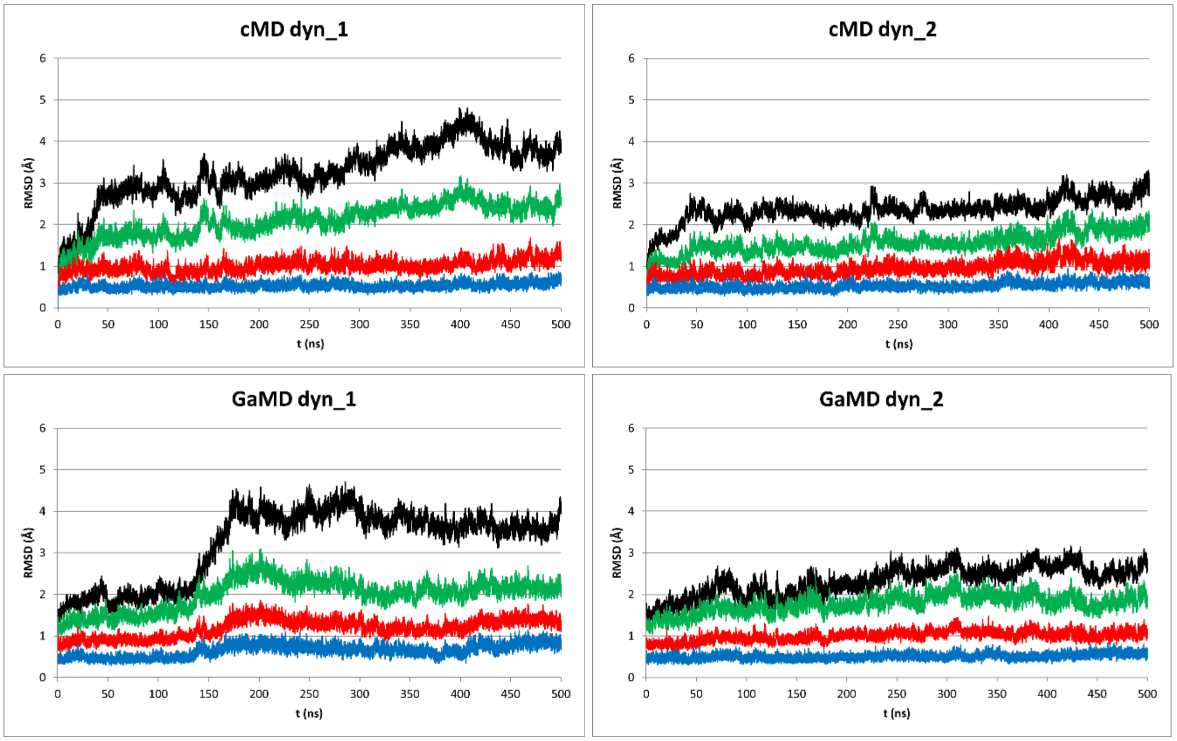

Figure S2. RMSD time evolution for each of the four different steps used in the determination of the protein residues presenting the smallest RMSF values, for both cMD and GaMD simulations runs. First iteration step is represented in color black, the second step is represented in, blue represents the third and magenta fourth steps respectively.

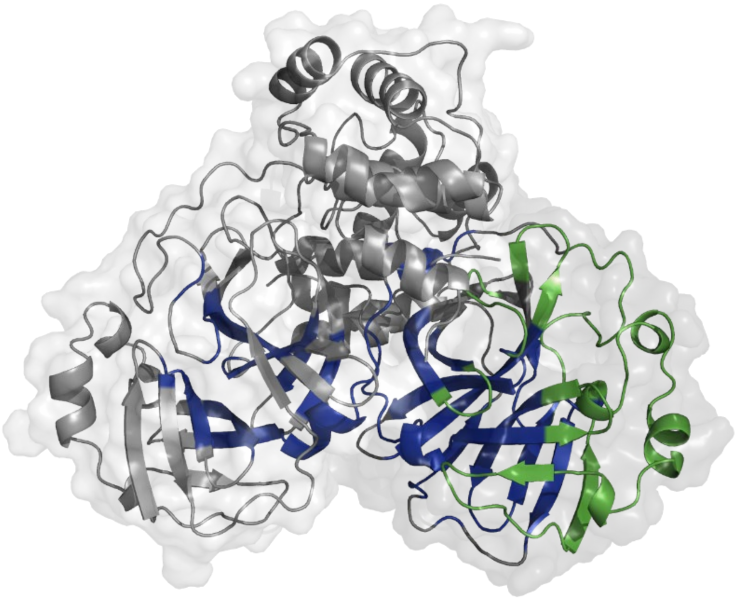

Figure S3. SARS-CoV-2 Mpro protease dimer representation. Residues colored in blue and green respectively represent the regions used in the superimposition of the structures (low mobility), and in the clustering and PCA analysis (high mobility).

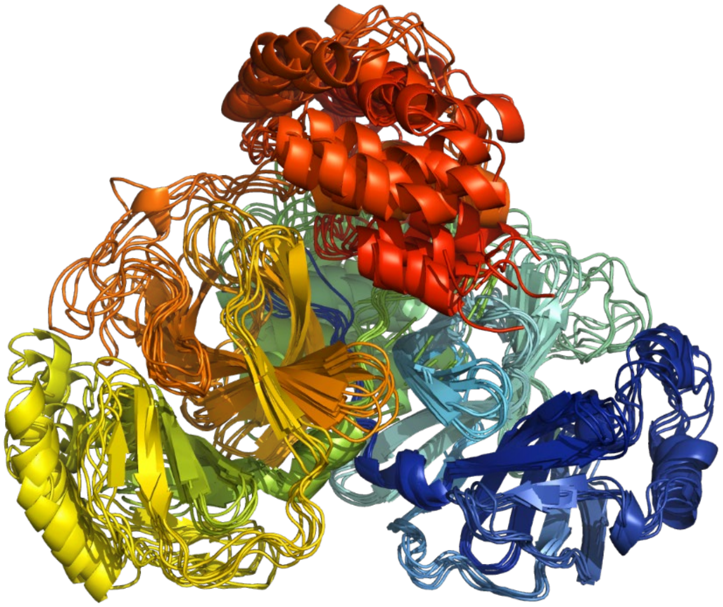

Figure S4. Superposition of the six representative structures of the SARS-CoV-2 Mpro protease dimer, identified from cMD and GaMD simulations. The Cα of the binding site presenting the lowest RMSF values have been selected as reference for the superposition.

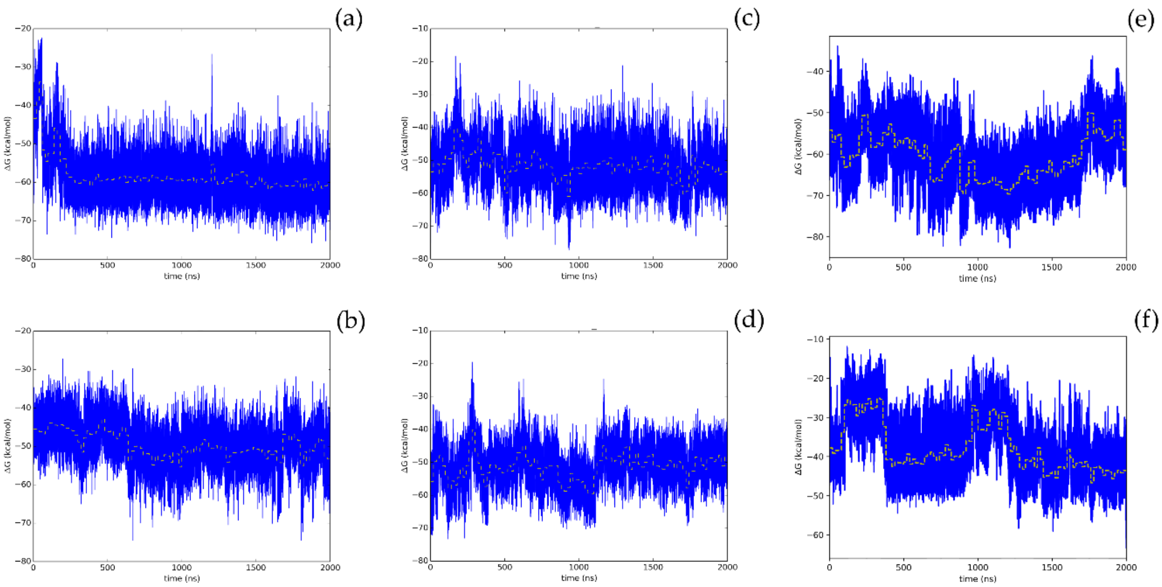

Figure S5. Time evolution of the MMGBSA binding free energy obtained for the full length extended molecular dynamic simulations of 2000 ns for the selected compounds. From left to right, binding energies corresponding to the first (a) and second (b) best binding sites identified for Hyperoside, Oligophenolic compounds SCH 644343 (c) and 644342 (d), Cinchonain Ia (e) and Cinchonain Ib (f). Average binding energies computed every 20 ns of the simulation are depicted in the graphs as a yellow dashed line.

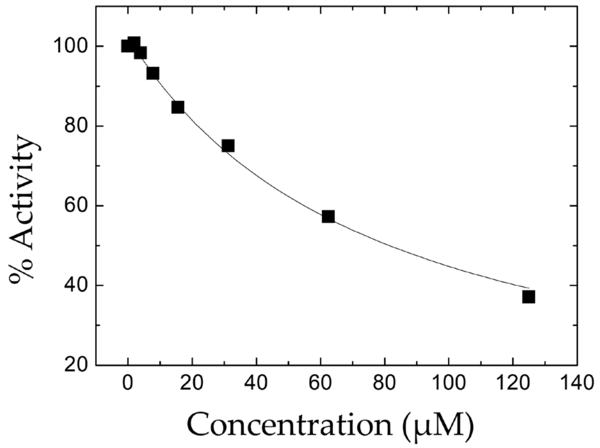

Figure S6. *In vitro* SARS-CoV-2 Mpro protease dimer inhibitory activity assay of Hyperoside natural compound selected from the multistep *In Silico* analysis performed. Serial dilutions were employed to evaluate the inhibitory effect in the FRET-based activity assay performed using a dual-fluorophore peptidic substrate and the recombinant SARS-CoV-2 M^{pro}. K_i, substrate concentration-independent inhibition constant, was computed by non-linear least-squares regression analysis following the description found in Materials and Methods section 4.5.3. IC_{50}, the half-maximal inhibitory concentration, was obtained by the application of a least squares fit to the normalized response curve defined by the inhibitor concentration.

Table S1. Free binding energy computed with the MMGBSA approach for 100 ns of cMD simulation of the evaluated compounds in complex with the SARS-CoV-2 M^{pro} main protease dimer. The energy of the best pose for each compound is depicted in the table. Units in Kcal/mol. Structures in bold, presenting the best and converged energy profiles, were selected for further analysis.

Natural Compound	cMD Representative 1	cMD Representative 2	cMD Representative 3	GaMD Representative 1	GaMD Representative 2	GaMD Representative 3
Hyperoside	-48.0	**-54.6**	-33.2	**-48.4**	**-46.6**	-39.2
Isoquercetin	-45.9	-37.4	-31.4	**-54.7**	-38.1	**-49.7**
Oligophenolic compound SCH 644343	-44.7	**-51.2**	-41.9	-51.3	**-67.5**	**-55.7**
Oligophenolic compound SCH 644342	-35.3	-39.3	**-51.0**	**-58.2**	-45.8	-50.1
Quercitrin	-30	**-51.9**	-37.0	-37.1	-32.1	
Quinovic acid glycoside	-26.5	-16.4	**-45.4**	**-39.5**	-17.6	-24.2
Cinchonain Ia	-32.4	**-37.9**	-32.3	-34.4	**-38.2**	**-60.7**
Cinchonain Ib	-20.8	**-37.5**	**-38.3**	-34.6	-21.9	**-33.8**
Loliolide	-8.8	-5.6	-23.7	-17.5	-26.3	-19.7

Table S2. Free binding energy computed with the MMGBSA approach for 500 ns of cMD simulation of the evaluated compounds in complex with the SARS-CoV-2 M^{pro} main protease dimer. Units in kcal/mol. Structures in bold, presenting the best and converged energy profiles, were selected for further analysis.

Natural Compound	cMD Representative 1	cMD Representative 2	cMD Representative 3	GaMD Representative 1	GaMD Representative 2	GaMD Representative 3
Hyperoside		**-59.3**		-44.7	**-46.2**	
Isoquercetin				**-40.8**		-42.9
Oligophenolic compound SCH 644343		**-52.3**			**-41.1**	**-38.3**
Oligophenolic compound SCH 644342			**-51.3**	**-52.4**		
Quercitrin		-32.9				
Quinovic acid glycoside			-36.4	-41.0		
Cinchonain Ia		**-32.5**			**-47.7**	**-57.4**
Cinchonain Ib		**-29.5**	**-42.9**			-41.8
Loliolide						

Table S3. Free binding energy computed with the MMGBSA approach for 1000 ns of cMD simulation of the evaluated compounds in complex with the SARS-CoV-2 M^{pro} main protease dimer. Units in kcal/mol. Structures in bold, presenting the best and converged energy profiles, were selected for further analysis.

Natural Compound	cMD Representative 1	cMD Representative 2	cMD Representative 3	GaMD Representative 1	GaMD Representative 2	GaMD Representative 3
Hyperoside		**-59.2**			**-55.5**	
Isoquercetin				**-44.0**		
Oligophenolic compound SCH 644343		**-52.4**			-36.4	**-38.3**
Oligophenolic compound SCH 644342			**-55.3**	-37.2		
Quercitrin						
Quinovic acid glycoside						
Cinchonain Ia		-33.80			**-45.20**	**-66.34**
Cinchonain Ib		-30.18	**-41.26**			**-38.18**
Loliolide						

Table S4. Free binding energy computed with the MMGBSA approach for 1500 ns of cMD simulation of the evaluated compounds in complex with the SARS-CoV-2 M^{pro} main protease dimer. Units in kcal/mol. Structures in bold, presenting the best and converged energy profiles, were selected for further analysis.

Natural Compound	cMD Representative 1	cMD Representative 2	cMD Representative 3	GaMD Representative 1	GaMD Representative 2	GaMD Representative 3
Hyperoside		**-60.3**			**-50.2**	
Isoquercetin				-37.8		
Oligophenolic compound SCH 644343		**-48.8**				-46.3
Oligophenolic compound SCH 644342			**-47.6**			
Quercitrin						
Quinovic acid glycoside						
Cinchonain Ia					-39.2	**-64.4**
Cinchonain Ib			-38.9			**-44.9**
Loliolide						

Table S5 Free binding energy computed with the MMGBSA approach for 2000 ns of cMD simulation of the evaluated compounds in complex with the SARS-CoV-2 M^{pro} main protease dimer. Units in kcal/mol. In bold, Hyperoside, the best binding energy and energetic profile exhibited during the complete simulation that was selected for *In Vitro* assays, exhibiting excellent results.

Natural Compound	cMD Representative 1	cMD Representative 2	cMD Representative 3	GaMD Representative 1	GaMD Representative 2	GaMD Representative 3
**Hyperoside**		**-60.1**			-53.3	
Isoquercetin						
Oligophenolic compound SCH 644343		-53.5				
Oligophenolic compound SCH 644342			-48.4			
Quercitrin						
Quinovic acid glycoside						
Cinchonain Ia						-59.0
Cinchonain Ib						-43.8
Loliolide						

Table S6. Binding free energy decomposition by residue computed for the last 100 ns of the complete 2 μs of cMD simulation of the two selected poses for Hyperoside in complex with the SARS-CoV-2 M^{pro} main protease dimer. Units in kcal/mol.

Protein Residue	BS 1	BS 2
K5	-1.8	
E14		-15.7
Y118		-1.9
S121		-1.9
P122		-3.3
S123		-3.2
K137	-3.3	
L286	-1.8	
L287	-1.0	
E288	-14.5	
D289	-14.5	
E290	-2.3	
G2′	-1.2	
F3′	-3.2	
R4′	-6.1	
F8′		-1.8
P9′		-2.8
K12′		-13.5
I152′		-3.0
D153′		-3.6
Y154′		-3.3
D155′		-1.8
L282′	-1.3	
G283′	-3.2	
S284′	-7.9	
A285′	-2.8	
L286′	-5.1	
F294′		-1.9
R298′		-1.4

‘Corresponding to residues of the chain B of the protease dimer.

Table S7. Most important Hydrogen Bonds stablished during the last 100 ns of the complete 2 μs of cMD simulation of the two selected poses for Hyperoside in complex with the SARS-CoV-2 M^{pro} main protease dimer.

System	Acceptor	Donor	Occupancy (%)
First Binding Site (-60.1 kcal/mol)	E288 (OE2)	Ligand (O2-H5)	99.6
	D289 (OD2)	Ligand (O10-H16)	96.7
	D289 (OD2)	Ligand (O9-H15)	96.4
	E288 (OE2)	Ligand (O3-H6)	94.7
	Ligand (O7)	S284' (OG-HG)	67.1
	Ligand (O11)	A285' (N-H)	64.6
	L287 (O)	Ligand (O8-H14)	17.3
	Ligand (O9)	D289 (N-H)	10.5
	Ligand (O4)	F3' (N-H)	8.1
	L282' (O-H7)	Ligand (O4-H7)	1.6
	Ligand (O2)	K5 (NZ-HZ2)	1.5
	Ligand (O2)	K5 (NZ-HZ1)	1.4
	Ligand (O2)	K5 (NZ-HZ3)	1.3
Second Binding Site (-53.3 kcal/mol)	E14 (OE1)	Ligand (O2-H5)	99.7
	E14 (OE1)	Ligand (O3-H6)	97.1
	D153' (O)	Ligand (O10-H16)	38.5
	D153' (O)	Ligand (O9-H15)	34.6
	Ligand (O3)	K12' (N-H)	13.7
	Ligand (O8)	K12' (NZ-HZ3)	11.4
	Ligand (O8)	K12' (NZ-HZ1)	9.2
	Ligand (O8)	K12' (NZ-HZ2)	8.7
	Ligand (O7)	K12' (NZ-HZ3)	6.9
	Ligand (O7)	K12' (NZ-HZ1)	5.6
	Ligand (O7)	K12' (NZ-HZ2)	5.1
	Y154' (O)	Ligand (O10-H16)	4.0
	D295' (OD1)	Ligand (O4-H7)	3.3
	P11' (O)	Ligand (O4-H6)	2.4
	D155' (OD2)	Ligand (O4-H15)	1.5
	Ligand (O5)	Y118 (O-H)	1.4
	S121 (OG)	Ligand (O4-H19)	1.3
	Ligand (O11)	K12' (NZ-HZ3)	1.0
	Ligand (O11)	K12' (NZ-HZ1)	1.0

'Corresponding to residues of the chain B of the protease dimer.

Table S8. Isomeric SMILES of the bioactive compounds selected from the literature search.

Natural Compound	SMILE
THHY	C#CCCCCCCCCCCCC(CC(CO)O)O †
Isoquercetin	C1=CC(=C(C=C1C2=C(C(=O)C3=C(C=C(C=C3O2)O)O)O[C@H]4[C@@H]([C@H]([C@@H]([C@H](O4)CO)O)O)O)O)O
Hyperoside	C1=CC(=C(C=C1C2=C(C(=O)C3=C(C=C(C=C3O2)O)O)O[C@H]4[C@@H]([C@H]([C@H]([C@H](O4)CO)O)O)O)O)O
22α-hydroxy-12-en-3-oxo-29-oic acid	C[C@]12CCC(=O)C(C1CC[C@@]3([C@@H]2CC=C4[C@]3(CC[C@@]5([C@@H]4CC(CC5O)(C)C(=O)O)C)C)C)(C)C
Quercetin	C1=CC(=C(C=C1C2=C(C(=O)C3=C(C=C(C=C3O2)O)O)O)O)O †
Quercitrin	C[C@H]1[C@@H]([C@H]([C@H]([C@@H](O1)OC2=C(OC3=CC(=CC(=C3C2=O)O)O)C4=CC(=C(C=C4)O)O)O)O)O
Oligophenolic Compound SCH 644343	C1C(OC2=CC(=CC(=C2C1=O)O)OC3C(C(C4C(O3)COC(=O)C5=CC(=C(C(=C5C6=C(C(=C(C=C6C(=O)O4)O)O)O)O)O)O)O)O)C7=CC=CC=C7 †
Oligophenolic Compound SCH 644342	C1C2C(C(C(C(O2)OC3=CC(=C(C(=C3)O)C(=O)CCC4=CC=CC=C4)O)O)O)OC(=O)C5=CC(=C(C(=C5C6=C(C(=C(C=C6C(=O)O1)O)O)O)O)O)O †
LOLIOLIDE	C[C@@]12C[C@H](CC(C1=CC(=O)O2)(C)C)O
Speciophylline	C[C@H]1[C@@H]2CN3CC[C@@]4([C@H]3C[C@@H]2C(=CO1)C(=O)OC)C5=CC=CC=C5NC4=O
Mitraphylline	C[C@H]1[C@H]2CN3CC[C@]4([C@@H]3C[C@@H]2C(=CO1)C(=O)OC)C5=CC=CC=C5NC4=O
Uncarine-F	C[C@H]1[C@@H]2CN3CC[C@]4([C@H]3C[C@@H]2C(=CO1)C(=O)OC)C5=CC=CC=C5NC4=O
Quinovic acid glycoside	C[C@]12CC[C@@H]([C@@]([C@@H]1CC[C@@]3([C@@H]2CC=C4[C@]3(CC[C@@]5([C@H]4CC(CC5)(C)C)C(=O)O[C@H]6[C@@H]([C@H]([C@@H]([C@H](O6)CO)O)O)O)C)C)(C)CO)O
Cinchonain Ia	C1[C@@H]([C@@H](CC2=C1C3=C(C=C2O)OC(=O)C[C@@H]3C4=CC(=C(C=C4)O)O)O)C5=CC(=C(C=C5)O)O
Cinchonain Ib	C1[C@@H]([C@@H](CC2=C1C3=C(C=C2O)OC(=O)C[C@H]3C4=CC(=C(C=C4)O)O)O)C5=CC(=C(C=C5)O)O

† Canonical SMILES were used instead.

Table S9. Physicochemical properties of Hyperoside.

Formula	MW (g/mol)		HBDs	HBAs	RB	MR	TPSA
C21H20O12	-54.6		8	12	-4	110.16	210.51

MW (Molecular weight); HBDs (Number of hydrogen bond donors); HBAs (Number of hydrogen bond acceptors); RB (Rotatable bonds); MR (Molar refractivity in m3/mol); TPSA(Topological polar surface area in Å).

Table S10. Medicinal chemistry, drug-likeness, pharmacokinetics, hydrophilicity, and lipophilicity properties of Hyperoside.

Medicinal Chemistry	***Drug-likeness***		***Pharmacokinetics***		***Water Solubility***	***Lipophilicity***
**SA**	**Lipinski**	**BA**	**GIA**	**BBBP**	**Log S**	**Log $P_{o/w}$**
C21H20O12	NO (2 violations)	0.17	Low	NO	-3.04 †	-0.38 ‡

SA (Synthetic accessibility from 1-10; BA (Bioavailability); GIA (Gastrointestinal absorption); BBBP (Blood-brain barrier permeability); LogS (water solubility); Log $P_{o/w}$ (Octanol/water partition coefficient).

† Experimental S = 27820 mg/L at 25 °C (HMDB 5.0: the Human Metabolome Database for 2022).

‡ Experimental LogP = - 0.111 (HMDB 5.0: the Human Metabolome Database for 2022).

File S1. File input for the QVina software.

```
center_x = 40.572
center_y = 41.244
center_z = 64.753
size_x = 37.5
size_y = 45.0
size_z = 41.25
cpu = 4
exhaustiveness = 8
num_modes = 15
energy_range = 3.0
```

File S2. File to run CPPTRAJ to obtain the top 10 PCs for the full conventional MD trajectory.

```
$AMBERHOME/bin/cpptraj    NoWat.PROTEASED.top  > PCA_cMD.out << EOF
trajin   ./rmds_cMD/proteaseD_cMD_NoWat.nc   1 last 1
projection Proj_pca  modes ./PROTD_cMD_GaMD.pev  out  PCA_cMD.ppj beg 1 end 10 :21-26,41-84,118-121,136-143,165-171,179-193@CA
run
EOF
```
